# Self-efficacy and self-rated oral health among pregnant aboriginal Australian women

**DOI:** 10.1186/1472-6831-14-29

**Published:** 2014-04-02

**Authors:** Lisa M Jamieson, Eleanor J Parker, Kaye F Roberts-Thomson, Herenia P Lawrence, John Broughton

**Affiliations:** 1 Australian Research Centre for Population Oral Health, University of Adelaide, Adelaide, SA 5005, Australia; 2 Otago University School of Dentistry, Dunedin, New Zealand; 3 Toronto University School of Dentistry, Toronto, ON, Canada

## Abstract

**Background:**

Self-efficacy plays an important role in oral health-related behaviours. There is little known about associations between self-efficacy and subjective oral health among populations at heightened risk of dental disease. This study aimed to determine if low self-efficacy was associated with poor self-rated oral health after adjusting for confounding among a convenience sample of pregnant women.

**Methods:**

We used self-reported data from 446 Australian women pregnant with an Aboriginal child (age range 14–43 years) to evaluate self-rated oral health, self-efficacy and socio-demographic, psychosocial, social cognitive and risk factors. Hierarchical entry of explanatory variables into logistic regression models estimated prevalence odds ratios (POR) and 95% confidence intervals (95% CI) for fair or poor self-rated oral health.

**Results:**

In an unadjusted model, those with low self-efficacy had 2.40 times the odds of rating their oral health as ‘fair’ or ‘poor’ (95% CI 1.54–3.74). Addition of socio-demographic factors attenuated the effect of low self-efficacy on poor self-rated oral health by 10 percent (POR 2.19, 95% CI 1.37–3.51). Addition of the psychosocial factors attenuated the odds by 17 percent (POR 2.07, 95% CI 1.28–3.36), while addition of the social cognitive variable fatalism increased the odds by 1 percent (POR 2.42, 95% CI 1.55–3.78). Inclusion of the behavioural risk factor ‘not brushing previous day’ attenuated the odds by 15 percent (POR 2.11, 95%CI 1.32–3.36). In the final model, which included all covariates, the odds were attenuated by 32 percent (POR 1.80, 95% CI 1.05, 3.08).

**Conclusions:**

Low self-efficacy persisted as a risk indicator for poor self-rated oral health after adjusting for confounding among this vulnerable population.

## Background

Self-efficacy has been described as one’s confidence in his or her ability to behave in ways to produce a desirable outcome [1]. In the dental setting, relatively little research exists on self-efficacy. Klepac et al. [2] presented evidence that associated dental anxiety with low self-efficacy regarding ability to tolerate tooth pain. Similarly, Kent and Gibbons [3] demonstrated that those who are more anxious are less confident about their ability to control their fear-related emotions regarding dentistry. Low dental self-efficacy of carers was associated with higher caries levels among Head Start children in the United States [4], while parental self-efficacy was found to be the strongest predictor of children’s brushing habits among pre-schoolers [5]. In a population sample of low-income African Americans, Finlayson and colleagues [6] reported that maternal self-efficacy was a predictor of child brushing.

Bandura [1] referred to four sources of self-efficacy. ‘Enactive mastery’ pertains to the experience of actual accomplishment and the subsequent increase in confidence that results. ‘Verbal persuasion’ is the notion that other people can give encouragement or otherwise make the case for increased competence. ‘Vicarious experience’ refers to the increase in confidence that sometimes results when people see a comparable other person coping successfully in a given situation. Finally, Bandura included ‘physiological state’ as a source of self-efficacy, reasoning that the experience of being less provoked than expected should increase confidence in a stressful circumstance.

Evidence suggests that psychosocial determinants help mitigate the effect of self-efficacy on health outcomes [7,8]. Relevant psychosocial determinants in regards to the efficacy/health relationship include perceived stress, sense of control, social support and perceived standing in society [9], while fatalism is a key component of Bandura’s social cognitive theory relating to self-efficacy and health [10].

Although low parental self-efficacy has been associated with adverse dental outcomes among children, there has been little documented evidence of the role of self-efficacy in adult oral health outcomes. Even less has been reported on groups known to be at high risk of dental disease. Aboriginal Australians [11] and pregnant women [12] are both groups at risk of dental disease. The purpose of this investigation was to determine if low self-efficacy was associated with poor self-rated oral health, a recognised proxy marker of both clinical oral health status [13] and oral health-related quality of life [14], among a convenience sample of Australian women pregnant with an Aboriginal child.

## Methods

### Study data and design

Participants were 446 women pregnant with an Aboriginal child in South Australia, Australia, who were part of a randomised controlled trial involving prevention of early childhood caries. Data used in this paper were thus cross-sectional and from a convenience sample. The participation rate was 100 percent and there were no incomplete responses (due to questionnaire data being collected via interview. Written informed consent was received). Participants represented around one-third of those who were eligible for the study during the study period (Feb 2011 to May 2012). We were unable to ascertain if our participants differed in significant ways to the source population due to the lack of Census, or other data, specifically pertaining to women pregnant with Aboriginal children. Participants were recruited from a range of sources including referrals from Aboriginal groups, community services and hospitals. The study received approval from the University of Adelaide Human Research Ethics Committee, the Aboriginal Health Council of South Australia, the Government of South Australia and the Human Research Ethics Committees of participating South Australian hospitals.

### Dependent variable

Poor self-rated oral health was defined as a response of ‘fair’ or ‘poor’ to the question: ‘How do you think your dental health is?’ Other options included ‘excellent’, ‘very good’ or ‘good’.

### Independent variables

Self-efficacy was based on an instrument developed by Finlayson and colleagues [15]. It was measured using a 6-item scale, asking participants to indicate how confident they feel about their ability to brush their teeth at night when they were: (1) under a lot of stress; (2) depressed; (3) anxious; (4) feeling that they were too busy; (5) tired or; (6) worried about other things in their life. The four response options ranged from ‘very confident’ to ‘not at all confident’. The possible score range is 0 to 24, with high scores indicating high self-efficacy. Alpha was 0.91. Self-efficacy was dichotomised based on a median split, with low self-efficacy pertaining to scores of 0 to 11 and high self-efficacy pertaining to scores of 12+.

The socio-demographic factors included age, education, income and ownership of a means-tested Government Health Care Card. A means-tested Government Health Care Card is provided to individuals on low income in Australia and enables them to have cheaper health care services and less expensive medicines. The age range of the sample was 14 to 43 years (mean 24.9 years, sd = 5.9). Age was dichotomised into ‘14 to 24 years’ and ‘25 years+’. Education was dichotomized into ‘high school or less’ or ‘trade/technical or University’, while Income was dichotomized into ‘Job’ or ‘Centrelink’ (welfare). ‘Centrelink’ is the Australian agency which provides welfare payments to those who are unemployed. Because such a small proportion of respondents earned their income from non-welfare means, the ‘job’ category included all forms of paid employment.

The psychosocial-related factors included stress, control, social support and subjective social standing. Stress was measured by the Perceived Stress Scale [16], which evaluates the frequency that people appraise situations as threatening and their appraised capacity to cope with threatening situations. There are 14 items in total, with five response options ranging from ‘not at all’ to ‘very often’. The possible score range is 0 to 56, with high scores indicating high stress. Alpha was 0.75. Stress was dichotomised, with low stress reflecting scores of 0 to 27 and high stress reflecting scores of 28+.

Control was assessed by the Sense of Personal Control scale [17], which comprises two dimensions referred to as ‘personal mastery’ and ‘perceived constraint’. The five response options for the 12 items range from ‘strongly disagree’ to ‘strongly agree’, with high scores indicating high control. The possible score range is 0 to 48 and alpha was 0.83. Control was dichotomised, with low control reflecting scores of 0 to 34 and high control reflecting scores of 35+.

Social support was assessed by four items, each designed to evaluate one of four dimensions of social support as theorised by House [18]. The dimensions include emotional, appraisal, instrumental and informational support. The five response options include ‘strongly agree’ to ‘strongly disagree’, with high scores indicating high social support. The possible score range is 0 to 16 (high scores equal high social support) and alpha was 0.86. Social support was dichotomised into 0 to 13 for low social support and 14+ for high social support.

Subjective Social Standing was assessed by the MacArthur Scale of Subjective Social Status [19]. The scale consists of a 10-rung ladder visual analogue scale on which participants rank themselves relative to others in their community (‘place in society’). Mean scores are calculated, with the possible range being 0 to 10. Subjective Social Standing was dichotomised, with low subjective social standing categorised as 0 to 5 and high subjective social support categorised as 6+.

The social cognitive factor included oral health-related fatalism and was based on an instrument developed by Finlayson and colleagues [15]. It was measured using a 3-item scale, asking participants to indicate their level of agreement with three oral health scenerios including pain, tooth loss and child dental caries. The five response options ranged from ‘strongly disagree’ to ‘strongly agree’. The possible score range is 0 to 12, with high scores indicating high fatalism. Alpha was 0.83. Fatalism was dichotomised, with low fatalism pertaining to scores of 0 to 8 and high fatalism pertaining to scores of 9+.

The risk behaviour included the question ‘did you brush your teeth yesterday?’, with response options including ‘yes’ or ‘no’.

### Analytic methods

**Figure 1:**
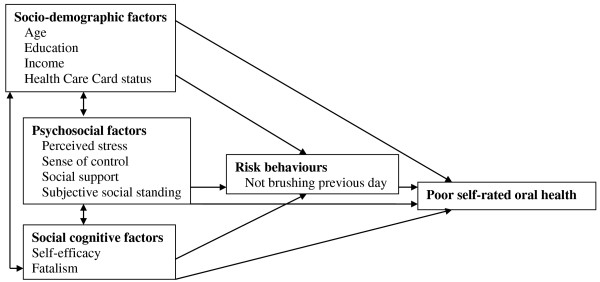
Postulated low self-efficacy and poor self-rated oral health pathway for women expecting Aboriginal babies.

Complete data were available for 446 participants. Correlation tests confirmed the existence of weak associations among the primary exposure (self-efficacy) and covariates (Pearson’s correlation coefficient range 0.1–0.4). No variables needed to be excluded due to collinearity. In bivariate analyses, prevalences and corresponding confidence intervals and p-values were generated by the ‘cross-tabulations’ analytical approach in SPSS. Blocks of explanatory variables were entered into a binary logistic regression model in six steps, as predicated by our conceptual model (Figure 1). The dependent variable of these models was self-rated oral health ‘fair’ or ‘poor’. Self-efficacy was entered in Model 1, with the main effect presented as a prevalence odds ratio (POR) with its 95% confidence interval (95% CI). The four socio-demographic factors were entered into Model 2, four psychosocial factors entered into Model 3, fatalism entered into Model 4 and the risk behavior entered into Model 5. The final model (Model 6) comprised all factors. This order of model-building was chosen so that individual effects of the domains represented in the conceptual model (socio-demographic factors, psychosocial factors, social cognitive factors and risk behaviours) could be assessed in relation to the outcome measure (poor self-rated oral health) before all variables were analysed in the final model. It is important to note that the final model was built based on apriori selection of covariates according to the conceptual model (Figure 1) as opposed to covariate selection based upon bivariate statistics. The degree of attenuation was calculated by the 1 - (ln(adjusted OR)/ln(unadjusted OR) formula. (Brotman [20]). We need to be clear that these estimates do not reflect risk. Data were analysed using IBM SPSS Statistics version 20.

## Results

Just over half the participants (55 percent) rated their oral health as ‘fair’ or ‘poor’ (Table 1). Almost two-thirds (63 percent) were categorized as having ‘low’ self-efficacy. Just over half (52 percent) the participants were aged 14 to 24 years, 72 percent had achieved high school or less as their highest education attainment, 86 percent had welfare-based income and 82 percent owned a health care card. Over one-third (37 percent) were categorized as having high stress, while 62 percent had a low sense of control. Around 58 percent reported low social support and 56 percent reported low subjective social standing. Around 39 percent of participants reported high levels of fatalism and one quarter (25 percent) had not brushed their teeth the previous day. A higher proportion of participants who reported low self-efficacy had lower levels of self-rated oral health, higher stress, lower sense of control, lower perceived social support, lower subjective social standing and did not brush the previous day (Table 1). Fair or poor self-rated oral health was associated with low self-efficacy, high school or less education attainment, high stress, low sense of control, low social support, low subjective social standing and not brushing the previous day.

**Table 1:** Frequencies, prevalences and unadjusted odds ratios for low self-efficacy and poor self-rated oral health

	**Frequency (95% CI)**	**Prevalence low self-efficacy (95% CI)**	**Unadjusted odds ratio (95% CI)**	**Prevalence poor self-rated oral health (95% CI)**	**Unadjusted odds ratio (95% CI)**
Self-rated oral health					
Excellent, very good or good	45.7 (41.1-50.4)	52.2 (44.0-60.0)*	0.42 (0.27-0.65)*	-	-
Fair or poor (ref)	54.3 (49.6-58.9)	72.4 (65.6-78.3)	1.00	-	-
Self-efficacy					
Low (0–11)	63.3 (58.1-68.2)	-	-	62.9 (56.3-69.0)*	2.40 (1.54-3.74)*
High (12+; ref)	36.7 (31.8-41.9)	-	-	41.4 (33.2-50.1)	1.00
Age					
14 to 24 years	52.2 (47.5-57.0)	60.8 (53.2-68.0)	0.80 (0.51-1.25)	50.7 (44.1-57.2)	0.76 (0.52-1.12)
25+ years (ref)	47.8 (43.0-52.5)	66.1 (58.5-72.9)	1.00	57.4 (50.5-64.1)	1.00
Education					
High school or less	71.6 (67.2-75.6)	65.3 (59.2-71.0)	1.41 (0.87-2.27)	56.5 (50.9-61.8)*	1.43 (0.94-2.16)
Trade or University (ref)	28.4 (24.4-32.8)	57.3 (47.2-66.8)	1.00	47.6 (39.0-56.4)	1.00
Income					
Job	14.1 (11.1-17.6)	68.0 (53.9-79.4)	1.28 (0.68-2.44)	43.5 (31.8-56.1)	0.61 (0.35-1.04)
Centrelink (ref)	85.9 (82.4-88.9)	62.3 (56.6-67.6)	1.00	55.9 (50.9-60.9)	1.00
HCC status					
Yes	82.2 (78.3-85.6)	62.9 (57.1-68.4)	0.85 (0.47-1.54)	55.9 (50.7-61.0)	1.52 (0.93-2.50)
No	17.8 (14.4-21.7)	66.7 (53.9-77.4)	1.00	45.5 (34.7-56.7)	1.00
Perceived stress					
Low (0–27)	63.4 (58.8-67.9)	53.9 (47.0-60.6)*	0.39 (0.24-0.62)*	47.8 (41.9-53.8)*	0.49 (0.33-0.73)*
High (28+)	36.6 (32.1-41.2)	75.2 (67.1-81.8)	1.00	65.2 (57.4-72.2)	1.00
Sense of control					
Low (0–34)	62.1 (57.4-66.5)	72.7 (66.3-78.2)*	3.13 (1.96-5.00)*	59.6 (53.6-65.3)*	1.73 (1.17-2.56)*
High (35+)	37.9 (33.5-42.6)	46.0 (37.4-54.8)	1.00	46.1 (38.6-53.7)	1.00
Social support					
Low (0–13)	58.3 (53.7-62.9)	69.3 (62.8-75.2)*	1.96 (1.25-3.03)*	59.8 (53.7-65.7)*	1.72 (1.17-2.51)*
High (14+)	41.7 (37.1-46.3)	53.7 (45.2-61.9)	1.00	46.5 (39.4-53.7)	1.00
Subjective social standing					
Low (0–5)	55.7 (50.9-60.3)	72.2 (65.4-78.0)*	2.56 (1.61-4.00)*	61.7 (55.3-67.6)*	1.84 (1.25-2.71)*
High (6+)	44.3 (39.7-49.1)	50.3 (42.2-58.4)	1.00	46.6 (39.6-53.7)	1.00
Fatalism					
Low (0–8)	61.5 (56.9-65.9)	61.6 (54.9-67.8)	0.82 (0.52-1.28)	54.6 (48.6-60.4)	1.01 (0.69-1.48)
High (9+)	38.5 (34.1-43.1)	66.2 (57.7-73.7)	1.00	54.4 (46.8-61.7)	1.00
Brush yesterday					
Yes	75.1 (70.8-79.0)	55.5 (49.3-61.5)*	0.22 (0.12-0.43)*	47.8 (42.2-53.3)*	0.45 (0.29-0.72)*
No (ref)	24.9 (21.0-29.2)	84.8 (75.1-91.2)	1.00	67.0 (57.5-75.3)	1.00

*P < 0.05.

In an unadjusted multivariable model, those with low self-efficacy had nearly two and a half times the odds of rating their oral health as ‘fair’ or ‘poor’ (Table 2, Model 1). Addition of socio-demographic factors to the self-efficacy variable attenuated the effect of low self-efficacy on poor self-rated oral health by 10 percent (Table 2, Model 2). Addition of the psychosocial factors to the self-efficacy variable attenuated the odds by 17 percent (Table 2, Model 3), while addition of the social cognitive variable fatalism to the self-efficacy variable increased the odds by 1 percent (Table 2, Model 4). Inclusion of the behavioural risk factor ‘not brushing previous day’ to the self-efficacy variable attenuated the odds by 15 percent (Table 2, Model 5). Low self-efficacy persisted as a risk indicator for poor self-rated oral health in the final model, which included all covariates. In this final model, the odds were attenuated by 32 percent (Table 2, Model 6).

**Table 2:** Multivariable models evaluating risk indicators for poor self-rated oral health among Australians expecting an Aboriginal child

	**Model 1 (POR, 95% CI)**	**Model 2 (POR, 95% CI)**	**Model 3 (POR, 95% CI)**	**Model 4 (POR, 95% CI)**	**Model 5 (POR, 95% CI)**	**Model 6 (POR, 95% CI)**
Self-efficacy						
Low (0–11)	2.40 (1.54-3.74)*	2.19 (1.37-3.51)*	2.07 (1.28-3.36)*	2.42 (1.55-3.78)*	2.11 (1.32-3.36)*	1.80 (1.05-3.08)*
High (12+; ref)	1.00	1.00	1.00	1.00	1.00	1.00
Age						
14 to 24 years	-	0.62 (0.39-0.98)*	-	-	-	0.62 (0.38-1.03)
25+ years (ref)	-	1.00	-	-	-	1.00
Education						
High school or less	-	1.23 (0.73-2.07)	-	-	-	1.36 (0.79-2.36)
Trade or University (ref)	-	1.00	-	-	-	1.00
Income						
Job	-	0.77 (0.33-1.76)	-	-	-	1.00 (0.41-2.42)
Centrelink (ref)	-	1.00	-	-	-	1.00
HCC status						
Yes	-	1.04 (0.48-2.23)	-	-	-	1.08 (0.48-2.43)
No	-	1.00	-	-	-	1.00
Perceived stress						
Low (0–27)	-	-	0.77 (0.46-1.29)	-	-	0.80 (0.46-1.40)
High (28+)	-	-	1.00	-	-	1.00
Sense of control						
Low (0–34)	-	-	0.99 (0.57-1.70)	-	-	1.13 (0.63-2.06)
High (35+)	-	-	1.00	-	-	1.00
Social support						
Low (0–13)	-	-	1.35 (0.82-2.23)	-	-	1.33 (0.78-2.26)
High (14+)	-	-	1.00	-	-	1.00
Subjective social standing						
Low (0–5)	-	-	1.34 (0.83-2.18)	-	-	1.12 (0.66-1.91)
High (6+)	-	-	1.00	-	-	1.00
Fatalism						
Low (0–8)	-	-	-	1.17 (0.75-1.82)	-	0.95 (0.56-1.60)
High (9+)	-	-	-	1.00	-	1.00
Brush yesterday						
Yes	-	-	-	-	0.74 (0.43-1.27)	0.88 (0.48-1.62)
No (ref)	-	-	-	-	1.00	1.00
-2 Log Liklihood	465.1	425.3	423.0	464.7	448.7	374.2
Nagelkerke R^2^	0.057	0.068	0.081	0.059	0.054	0.088

*P < 0.05.

## Discussion

Low self-efficacy was a risk indicator for poor self-rated oral health in this convenience sample of pregnant Aboriginal Australians; a group recognised as being of high risk of both dental caries and periodontal disease [11]. This association persisted even after adjusting for socio-demographic, psychosocial, social cognitive and behaviour covariates. In the final model, self-efficacy was the only variable that remained significant. To the best of our knowledge, this is the first time risk indicators for self-rated oral health in an Australian Aboriginal population have been reported and the first time an association between self-efficacy and self-rated oral health in any population has been established.

Due to the cross-sectional nature of our study, the findings cannot be considered to be causal. Indeed, it is possible that poor self-rated oral health may lead to feelings of low self-efficacy. The self-report nature of the data may have led to an under-estimation of these factors, with incorrect responses potentially given for any number of reasons (social desirability bias, difficulty understanding English, not comprehending question). However, we took great care with interviewing and, in any case, non-differential under-reporting would have resulted in more conservative estimates, meaning our findings are unlikely to be spurious. Evidence from the literature suggests, however, that self-efficacy is likely to be a driver of certain behaviours that lead to health outcomes, with clinical studies typically using self-efficacy as an antecedent to behaviour modification. In the dental field, McCaul and colleagues [21] reported that self-efficacy was significantly related to both retrospective and prospective self-monitored frequency of brushing and flossing among college students, while Tedesco et al. [22] found that linking self-efficacy variables to theory of reasoned action variables significantly increased the variance observed in brushing and flossing behaviours. In a study that compared psychological characteristics such as self-efficacy, locus of control and self-esteem in relation to oral health habits, dental caries and periodontal disease, Syrjala et al. [23] reported that only self-efficacy was associated with all dental outcomes. In regards to self-efficacy-based oral health interventions, Kakudate and colleagues [24] conducted a randomised controlled trial to compare effectiveness of an oral hygiene-based enhanced self-efficacy intervention with conventional oral hygiene instruction. The intervention group had improved plaque index scores, toothbrushing duration and frequency of inter-dental cleaning in comparison with the control group, leading the authors to conclude that the effectiveness of a behavioural intervention to enhance self-efficacy and promote oral health-related behavioural change was observed.

## Conclusion

The findings suggest that, in our study, the self-efficacy association with poor self-reported oral health exists beyond the tooth-brushing pathway. Other potential mechanisms linking self-efficacy and oral health status may include high-sugar diet, problem-based dental attendance or dental fear. Future studies would do well to include analyses of these additional factors.

Self-efficacy among Aboriginal Australians has not been extensively researched. However, the Australian Institute of Health and Welfare, who are involved in the conduct and publication of Australia’s National Aboriginal and Torres Strait Islander Health Surveys, suggests that social and emotional well-being concepts such as self-efficacy should be considered in future waves [25]. Our findings give some evidence that the role of self-efficacy in health outcomes such as self-rated oral health among Aboriginal populations may be under-appreciated, and that this association needs to be further explored. The convenience sample means the findings are not able to be generalised to other population groups, meaning further research among other populations, preferably utilising a longitudinal design, is required.

## Competing interest

The authors declare that they have no competing interests.

## Authors’ contributions

LMJ conceived of the study, participated in its design and co-ordination and drafted the manuscript. EJP made substantial contributions to the design of the study and interpretation of data. KRT made substantial contributions to the design of the study and interpretation of data. JSB was involved in drafting the manuscript or revising it critically for important intellectual content. HPL was involved in drafting the manuscript or revising it critically for important intellectual content. All authors read and approved the final manuscript.

## Pre-publication history

The pre-publication history for this paper can be accessed here:

http://www.biomedcentral.com/1472-6831/14/29/prepub
